# Measuring and evaluating progress towards Universal Health Coverage in China

**DOI:** 10.7189/jogh.11.08005

**Published:** 2021-05-01

**Authors:** Xiaoyun Liu, Ziyue Wang, Huan Zhang, Qingyue Meng

**Affiliations:** 1 China Centre for Health Development Studies, Peking University, Beijing, China; 2 Faculty of Epidemiology and Population Health, London School of Hygiene and Tropical Medicine, London, UK

## Abstract

**Background:**

This paper aims to develop a Chinese version of Universal Health Coverage (UHC) indices and to measure China’s progress towards UHC.

**Methods:**

Nineteen indicators were selected based on expert consultations to construct indices of accessibility and affordability to measure UHC. Data were drawn from health statistics yearbooks, nationally representative surveys, and health system reform surveillance. The index of accessibility includes absolute accessibility (to essential health services), relative accessibility (to hospital care) and people’s subjective perceptions. The index of affordability includes absolute affordability (the incidence of catastrophic health expenditure, CHE), relative affordability (the composition of health expenditure), and people’s subjective perceptions.

**Results:**

The indices of accessibility and affordability both showed steady increases over the 17 years considered. Absolute accessibility had the most significant improvement (from 23.6 in 2002 to 73.8 in 2018), while the index of relative accessibility decreased from 81.4 in 2002 to 67.3 in 2018. The index of absolute affordability decreased significantly from 46.6 in 2002 to 30.5 in 2010 and then exhibited an increasing trend afterwards, reaching 52.1 in 2018. The index of relative affordability continuously increased during the observation period, from 35.3 to 75.4.

**Conclusions:**

China has made great progress in increasing the accessibility and affordability of health services since the health system reforms in 2009. However, integrating primary health care and hospital care and containing escalating medical expenditure to further reduce patients’ financial burdens are key challenges for strengthening the Chinese health system.

Universal Health Coverage (UHC) is widely accepted as a health-related SDG. Measuring and evaluating progress towards UHC is a critical task globally and for individual countries. The World Health Organization (WHO) and its partners have developed indices to measure coverage of essential health services (SDG 3.8.1) and financial risk protection (SDG 3.8.2) [1,2]. Sixteen tracer indicators from four areas of health care were selected to calculate the index of essential health services using geometric means. A single indicator for catastrophic health expenditure (CHE) was used to measure financial risk protection.

These two global indices use straightforward approaches and internationally comparable data. They are greatly valuable for global monitoring and allow countries to use similar or modified formats to track UHC progress. However, since countries have different health system challenges and different health data availability, a uniform global index may not be able to reflect each country’s unique situation [3]. Countries and regions are encouraged to refine and tailor the UHC indices to their local circumstances.

China started an overall health system reforms in 2009 to address two key challenges in achieving UHC, namely, “difficulty in seeking medical services” and “high expenses related to medical service utilization” [4]. While these two challenges reflect well the UHC dimensions of “essential health services coverage” and “financial risk protection”, they also include features unique to the Chinese health system. First, accessibility includes both potential availability of health services (eg, sufficiency of health workers) and actual utilization of health services [5,6]. The accessibility challenge in China does not only concern the essential health care, but largely reflects complaints about the difficulties in accessing advanced hospital care [7]. Second, the concept of affordability has been framed into absolute measures related to poverty and relative measures that apply to the entire income spectrum. Catastrophic health expenditure has been widely used to measure absolute affordability. China’s health financing reform has made great efforts to improve the share of government investment in total health expenditures and to reduce patients’ out-of-pocket payment share. In addition to the objective measures of accessibility and affordability, peoples’ subjective perceptions of accessibility and affordability have been a key driving force in the health system reform. These key features of the Chinese health system should be considered in measuring China’s progress towards UHC.

This paper aims to develop a Chinese version of the UHC indices and to measure China’s progress towards UHC. The study will draw lessons from the WHO’s UHC indicators but will build China’s context into the indices. This exercise of developing country-specific UHC indicators will not only help policy makers monitor China’s progress towards achieving UHC but also set an example of tailoring UHC monitoring to a specific country context.

## METHODS

### Index component selection

Two indices for China’s UHC monitoring were developed, namely, the index of accessibility and the index of affordability.

In selecting the index components and indicators, the following criteria were followed. First, access to essential health services should cover both public health services (eg, maternal and childcare, essential vaccines/immunization services, and chronic diseases management, which are key indicators of the UHC framework developed by the WHO) and medical services, including primary health care (PHC) and hospital care. The affordability of health services should consider both patients’ actual financial burden (out-of-pocket payments and catastrophic health expenditure) and government’s efforts to reduce financial burden through investments in health. Additionally, public opinion on accessibility and affordability should be included in the measurement. These factors are key features of the current development of China’s health system. Second, quality and equity dimensions should be considered in selecting indicators. Third, most data used should be publicly available so that the indices can be validated by others and updated in the future. After drafting a list of components and indicators, we engaged in three rounds of expert consultations to refine the components and indicators. The experts consisted of academic researchers studying health systems, national health policy makers and health managers.

The index of accessibility has three components (**Table 1**). Absolute accessibility refers to access to essential health services. It has 9 indicators covering 4 areas of essential health services, namely, essential medical services, essential public health services, essential medicine, and basic health insurance. Relative accessibility refers to access to hospital care. This reflects the situation in China in which many patients bypass PHC and go directly to hospitals. The index of relative accessibility has 3 indicators, including the proportion of hospitalization service utilization within a county, the proportion of outpatient visits at the PHC level, and the proportion of patients not using hospitalization services when recommended. Subjective perceptions of accessibility are measured by patients’ satisfaction with outpatient and inpatient services.

**Table 1:** Components and indicators for constructing UHC indices in China

Index	Index component	Indicator	Definition/rational	Data source
**Accessibility**	**Absolute accessibility**	1. % of resident with access to the nearest health facilities within 15 min	Geographic access to essential health services	NHSS
		2. Number of physicians per 1000 population	Availability of health workforce	Health statistics yearbook
		3. % of physicians with bachelor’s degree or above*	Measuring quality of health workforce	Health statistics yearbook
		4. Number of general practitioners per 10 000 population	Availability of workforce for primary health care	Health statistics yearbook
		5. Number of outpatients visit per person per year	Utilization of outpatient services	Health statistics yearbook
		6. Annual hospitalization rate (%)	Utilization of inpatient services	Health statistics yearbook
		7. Coverage of essential public health services	Covering 15 items of essential public health services†	Health system reform surveillance, CHS, CNNHS, PEACE Project, CCDRFS
		8. % of PHC facilities equipped with essential medicine	Availability of essential medicine	Health system reform surveillance
		9. Coverage of basic health insurance schemes	Access to health insurance schemes	Health statistics yearbook
	**Relative accessibility**	10. % of hospitalization within the county	Reflecting national policy priority that essential services utilization should be within local county	Health system reform surveillance
		11. % of outpatient service utilization at PHC level	Reflecting national policy priority to attract more patients to use PHC services	Health statistics yearbook
		12. % of patients recommended but not using inpatient service	Reflecting patients’ access to inpatient survey	NHSS
	**Subjective perception on accessibility**	13. Patients’ satisfaction with outpatient services	Patients’ objective perception on accessibility to outpatient services	NHSS
		14. Patients’ satisfaction with inpatient services	Patients’ objective perception on accessibility to inpatient services	NHSS
**Affordability**	**Absolute affordability**	15. % of catastrophic health expenditure	Reflecting patients’ financial burden due to seeking health services	CFPS, NHSS.
		16. % of catastrophic health expenditure among low income group	Reflecting poor patients’ financial burden due to seeking health services	CFPS
	**Relative affordability**	17. % of medical expenses covered by health insurance	Reflecting health insurance’s role on reducing patients’ financial burden	Health system reform surveillance
		18. % of out of pocket payment in total health expenditure	Reflecting patients’ overall financial burden	Health statistics yearbook
		19. % of total health expenditure in GDP	Reflecting overall health financing	Health statistics yearbook
	**Subjective perception on affordability**	20. Patients’ satisfaction with outpatient services	Patients’ objective perception on affordability to outpatient services	NHSS
		21. Patients’ satisfaction with inpatient services	Patients’ objective perception on affordability to inpatient services	NHSS

UHC – universal health coverage, PHC – primary health care, GDP – gross domestic product, NHSS – National health services survey, CHS – China Hypertension Survey, CNNHS – China National Nutrition and Health Survey, PEACE – China Patient-Centered Evaluative Assessment of Cardiac Events Project, CCDRFS – China Chronic Disease and Risk Factors Surveillance, CFPS – China Family Panel Studies

*Due to the unique history of medical education in China, licensed doctors have various education backgrounds, including: 1. medical university, graduated with a bachelor’s degree of medicine (5 y of medical education after high school); 2. junior medical college (3 years of medical education after high school); 3. technical school (3 years of medical education after middle school).

†Including 4 indicators of maternal and child care (1. antenatal care coverage (at least four times during pregnancy), 2. health management coverage for children <3 years, 3. postpartum care coverage, and 4. physical examination coverage for children <5 years), 6 indicators of chronic diseases, mental health, and aging-health care (1. hypertension management rate, 2. type 2 diabetes management rate, 3. severe mental illness management rate, 4. health management coverage for older adults >65 years, 5. hypertension control rate, 6. type 2 diabetes control rate), and 5 immunization rates indicators (1. diphtheria-tetanus-pertussis (DTP3) immunization coverage <5 years, 2. bacillus Calmette-Guérin (BCG) immunization coverage <5 years, 3. measles immunization coverage <5 years, 4. poliomyelitis immunization coverage <5 years, 5. hepatitis B immunization coverage <5 years).

The index of affordability also has three components. Absolute affordability refers to patients’ real financial burdens due to seeking health services. This is measured by the incidence of catastrophic health expenditure (CHE). We also include the proportion of CHE among those with low income to add an equity perspective. Relative affordability refers to the composition of medical expenditure (government investment and out-of-pocket payments). It has three indicators: the percent of medical expenses covered by health insurance, the percent of out-of-pocket payments in total health expenditure, and the percent of total health expenditure in gross domestic product (GDP). Subjective perceptions of affordability are also measured by patients’ satisfaction with outpatient and inpatient services.

### Indicator data sources

Data for the 19 indicators were drawn from three sources: health statistics yearbooks, nationally representative surveys, and health system reform surveillance. Since all selected data are open access, ethical approval was not required.

The health statistics yearbooks contain routine monitoring data on human resources for health, health service utilization, and health outcomes at the national and provincial levels. Data from 2003 to 2018 were extracted and included in the analysis [8].

Data from various nationally representative surveys were included. Since 1993, the National Health Commission conducted national health services survey every five years. The national health services survey collected information on health needs and service utilization using a repeated cross-sectional study design [9,10]. Indicators for geographic access to the nearest health facilities, patients needing but not using inpatient services, and patients’ satisfaction with outpatient and inpatient services were extracted from the national health service survey reports from 2003 to 2018. Data on CHE among the whole population and the low-income subpopulation were drawn from the China Family Panel Studies (CFPS). CFPS is a nationally representative, annual longitudinal survey of Chinese communities, families, and individuals launched in 2010 by Peking University. Four rounds of CFPS data (2010, 2012, 2014, 2016) were analysed to obtain the CHE incidence. CHE before 2010 was calculated based on the national health services survey [10]. In addition, we obtained the data on chronic diseases management (calculated in the essential public health services data) from multiple nationally representative surveys: control rates for hypertension from the China Hypertension Survey, the China National Nutrition and Health Survey, and the China Patient-Centered Evaluative Assessment of Cardiac Events (PEACE) Million Persons Project [11-13]. Data on the blood glucose control rate among diabetes patients was from the China Chronic Disease and Risk Factors Surveillance study [4]. The indicators estimated from multiple data sources (CHE, hypertension) are comparable.

The National Health Commission has been gathering surveillance data to monitor the progress of the health system reforms. Data on the coverage of essential public health services (maternal and childcare, essential vaccines / immunization services), the percent of PHC facilities equipped with essential medicine, and the percent of medical expenses covered by health insurance are collected from this health system surveillance.

Longitudinal data from 2002 to 2018 were extracted to analyse changes in the trend towards UHC. Some indicators had missing values. For example, data on patients’ satisfaction with outpatient and inpatient services was drawn from the national health services survey, which was only available for four years (2003, 2008, 2013, and 2018). Missing values were estimated by interpolation and extrapolation based on linear regression [14]. Two indicators (availability of essential medicine and proportion of hospitalization within their counties) were not included in the final index calculation due to data availability of less than 25% at the time of analysis.

### Index construction

Before constructing the indices, all indicators were standardized, resulting in values ranging from 0 to 100 and with 100 as the target. Some indicators can be incorporated directly into the index without being standardized, for example, the percentage of residents with access to a health facility within 15 minutes (target value should be 100%). Other indicators need to be transformed based on a target value. These target values were drawn from either national policy targets or international standards. For example, the National Plan for a Healthy China 2030 set a target to have 3 physicians per 1000 people. This was used as the target value for this indicator. The transformed indicator value was equal to (*x*/3) × 100%. For the percentage of CHE, the minimum global value was 1%, and the maximum value was 28% in 2015 [2]. The transformed indicator value was set as (*x*-28%)/(1%-28%) × 100%. (see Appendix S1 of the **Online Supplementary Document** for the transformation formulas for all indicators.)

**Figure 1:**
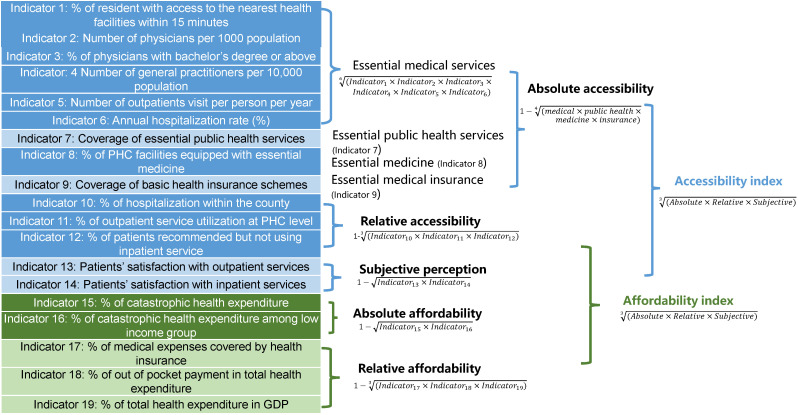
Calculation method for the Index of accessibility and Index of affordability.

We used geometric means to calculate the indices of accessibility and affordability. Geometric means favour equal coverage levels across services as opposed to higher coverage for some services at the expense of others and can increase the sensitivity of the UHC index to the very low value of individual indicators. Geometric means were frequently used to develop such index, mostly drawing inspiration from the Human Development Index. [15] The WHO/World Bank version of UHC index [2], and a few other studies on UHC indices [16,17] also used geometric means. This was a three-step process (**Figure 1**). First, individual indicators (1 to 5) were merged into an index of essential medical services by calculating the geometric means. Second, the index of essential medical services, essential public health services, essential medicine, and basic health insurance were converted into an index of absolute accessibility. Similarly, indices of relative accessibility, absolute and relative affordability, and patient perceptions of accessibility and affordability were calculated. Third, the index of accessibility was calculated based on absolute accessibility, relative accessibility, and patient perceptions. The index of affordability was calculated based on absolute affordability, relative affordability, and patient perceptions. The values of the accessibility and affordability indices ranged from 1 to 100, with 100 as the ideal target value.

### Statistical analysis

We performed sensitivity analyses under different scenarios to examine the robustness of our results. In scenario 1, the indices of accessibility and affordability were calculated from the original indicators, without using the step-by-step calculation process from individual indicators through sub-index to overall index. In scenario 2, we used the arithmetic mean to calculate the indices instead of using geometric means. In scenario 3, we only use the data available to us without any imputation. Finally, in scenario 4, indices were repeatedly calculated with one indicator removed from the calculation each time. The sensitivity analysis results are reported in Appendix S2 of the **Online Supplementary Document**.

We used single-group interrupted time-series (ITS) analysis to assess the association between the 2009 health system reform and the indices of accessibility and affordability. The analysis results are reported in Appendix S3 of the **Online Supplementary Document**.

Provinces in China have imbalanced progress in socioeconomic and health development. We calculated the indices of accessibility and affordability for 25 provinces in mainland China (Inner Mongolia, Hainan, Tibet, Qinghai, and Ningxia were excluded) with available data from 2018. The 25 provinces were compared in terms of accessibility and affordability. The analysis results are reported in Appendix S4 of the **Online Supplementary Document**.

### Ethical approval

Not required.

## RESULTS

### Changing trend of individual indicators

**Table 2** shows that the values of 14 of the 15 indicators improved, and only the proportion of outpatient visits at the PHC level decreased over time (from 94.2 in 2002 to 57.6 in 2018). Four indicators improved more than 40 points since 2002, including essential health insurance coverage (from 7.3 in 2002 to 96.3 in 2018), annual hospitalization rate (from 38.9 in 2002 to 100 in 2018), the health care reimbursement rate (from 16.0 in 2002 to 67.1 in 2018), and the number of outpatient visits per capita (from 32.2 in 2002 to 78.3 in 2018).

**Table 2:** Scores of all indicators of accessibility and affordability at the national level from 2002 to 2018 (full marks = 100)*

Indicators	2002	2003	2004	2005	2006	2007	2008	2009	2010	2011	2012	2013	2014	2015	2016	2017	2018
**Absolute accessibility**	**23.6**	**24.6**	**27.7**	**29.7**	**33.2**	**37.0**	**39.3**	**47.0**	**51.7**	**55.4**	**58.8**	**62.2**	**64.2**	**65.9**	**68.1**	**70.8**	**73.8**
% of resident with access to the nearest health facilities within 15 min	79.9‡	79.9	79.8‡	79.7‡	79.6‡	79.5‡	79.4	80.3‡	81.2‡	82.2‡	83.1‡	84.0	85.2‡	86.4‡	87.5‡	88.7‡	89.9
Number of physicians per 1000 population	39.0	40.7	41.7	41.3	42.7	43.3	45.0	47.7	49.0	49.7	52.7	55.7	58.0	61.3	64.0	68.0	72.0
% of physicians with bachelor’s degree or above	33.5	35.2‡	36.8‡	38.5	41.6‡	44.8‡	47.9‡	51.0	51.5	53.0‡	54.5	57.2	56.8‡	56.4	58.3	60.1	62.5
Number of general practitioners per 10 000 population	2.1‡	2.1‡	2.1‡	2.1‡	2.1‡	2.1‡	2.1‡	5.8‡	9.5‡	13.2‡	16.2	21.4	25.4	27.4	30.2	36.4	44.0
Number of outpatients visit per person per year	32.2	35.4	40.4	41.2	44.7	47.0	48.6	54.1	57.3	61.2	67.1	71.1	73.7	73.7	75.0	77.6	78.3
Annual hospitalization rate (%)	38.9	39.3	42.8	45.8	50.1	62.0	72.2	82.9	88.3	94.8	100	100	100	100	100	100	100
Coverage of essential public health services	46.1†	47.0†	48.3†	49.3†	50.0†	51.0†	50.6†	50.5†	54.5†	55.3†	55.6†	55.4†	56.1†	60.5†	65.5†	65.5†	65.4†
% of PHC facilities equipped with essential medicine	NA	NA	NA	NA	NA	NA	NA	NA	NA	NA	NA	NA	NA	92.3	84.9	NA	NA
Coverage of basic health insurance schemes	7.3	8.4	15.7	24.2	43.2	71.8	86.9	92.5	94.6	96.9	99.1	100	97.5	97.2	95.7	94.2	96.3
**Relative accessibility**	**81.4**	**80.5**	**80.1**	**79.6**	**79.1**	**78.6**	**75.5**	**78.0**	**78.9**	**78.2**	**77.6**	**77.7**	**75.0**	**73.3**	**70.9**	**69.2**	**67.3**
% of hospitalization within the county	NA	NA	NA	NA	NA	NA	NA	NA	NA	NA	NA	NA	NA	79.7	76.4	81.6	81.7
% of outpatient service utilization at PHC level	94.2‡	92.1‡	89.9‡	87.8‡	85.6‡	83.5‡	76.1	79.5	79.7	76.7	74.1	72.8	68.5	66.1	62.6	60.3	57.6
% of patients recommended but not using inpatient service	70.4‡	70.4	71.3‡	72.2‡	73.1‡	74.0‡	74.9	76.5‡	78.1‡	79.7‡	81.3‡	82.9	82.0‡	81.2‡	80.3‡	79.5‡	78.6
**Subjective perception on accessibility & affordability**	**50.3**	**50.3**	**51.7**	**53.2**	**54.6**	**55.9**	**57.3**	**60.2**	**63.1**	**65.9**	**68.8**	**71.7**	**72.9**	**74.0**	**75.2**	**76.3**	**77.5**
Patients’ satisfaction with outpatient services	57.1‡	57.1	57.4‡	57.8‡	58.1‡	58.5‡	58.8	62.3‡	65.9‡	69.4‡	73.0‡	76.5	77.2‡	77.9‡	78.6‡	79.3‡	80.0
Patients’ satisfaction with inpatient services	44.3‡	44.3	46.6‡	48.9‡	51.2‡	53.5‡	55.8	58.1‡	60.4‡	62.6‡	64.9‡	67.2	68.8‡	70.3‡	71.9‡	73.4‡	75.0
**Absolute affordability**	**46.6**	**46.6**	**44.6**	**42.7**	**40.7**	**38.7**	**36.7**	**34.1**	**30.5**	**38.1**	**37.8**	**43.2**	**48.5**	**50.3**	**52.1**	**52.1**	**52.1**
% of catastrophic health expenditure	58.5‡	58.5	57.2‡	55.9‡	54.5‡	53.2‡	51.9	53.2‡	52.4	55.9	55.2	60.5‡	65.8	68.2‡	70.6	70.6‡	70.6‡
% of catastrophic health expenditure among low income group	37.0‡	37.0	34.8‡	32.6‡	30.4‡	28.2‡	25.9	21.8‡	17.7	25.9	25.9	30.8‡	35.7	37.0‡	38.4	38.4‡	38.4‡
**Relative affordability**	**35.3**	**35.8**	**39.4**	**42.3**	**45.2**	**48.3**	**52.5**	**56.8**	**58.8**	**61.3**	**64.4**	**67.0**	**69.0**	**72.2**	**74.2**	**74.5**	**75.4**
% of medical expenses covered by health insurance	16.0‡	16.0	20.6‡	25.2‡	29.9‡	34.5‡	39.1	42.7‡	47.8‡	52.1‡	57.5‡	62.9	64.8	65.8	67.5	67.1	67.1
% of out of pocket payment in total health expenditure	51.0	53.3	56.0	57.7	61.2	67.5	71.9	75.5	78.1	78.7	79.3	79.8	82.1	85.4	86.0	86.0	86.2
% of total health expenditure in GDP	53.7	54.0	52.9	52.1	50.6	48.3	51.3	56.7	54.6	56.1	58.6	60.0	61.8	67.1	70.2	71.7	74.1
**Index of accessibility**	**45.9**	**46.4**	**48.6**	**50.1**	**52.3**	**54.6**	**55.4**	**60.4**	**63.6**	**65.9**	**68.0**	**70.3**	**70.5**	**71.0**	**71.4**	**72.1**	**72.8**
**Index of affordability**	**45.4**	**45.7**	**46.6**	**47.1**	**47.5**	**47.8**	**48.4**	**49.4**	**49.1**	**54.5**	**56.2**	**60.5**	**63.7**	**65.6**	**67.2**	**67.5**	**68.0**

PHC – primary health care, N/A – not available

*Note: The indicators 8 and 10 did not have widespread data availability and were excluded from the index calculations.

†Different methodology.

‡Estimated value.

The five indicators with the lowest values in 2018 were the number of general practitioners (44.0), the number physicians with bachelor’s degrees or higher (62.5), the incidence of catastrophic health expenditure in the low-income sub-population (38.4), the proportion of outpatient visits at the PHC level (57.6), and essential public health services coverage (65.4).

### Index of accessibility

**Figure 2:**
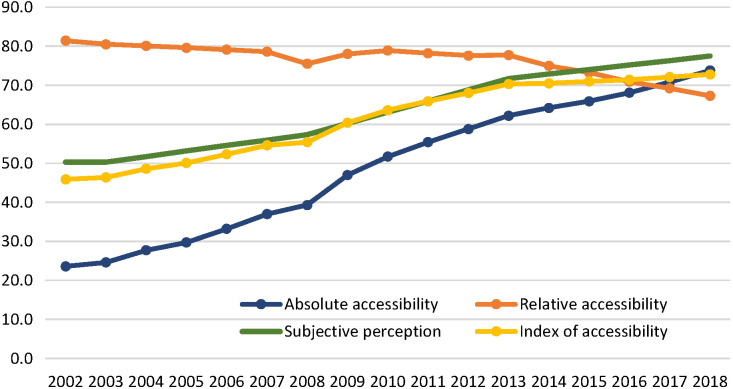
Change in index of accessibility in China (2002-2018).

Among the three components of accessibility, absolute accessibility had the most significant improvement. In 2002, the absolute accessibility index was 23.6. It increased to 47.0 by 2009. After the health care reforms were implemented in 2009, the absolute accessibility index further increased to 73.8. The index of subjective perceptions of accessibility has also improved, from 50.3 in 2002 to 60.2 in 2009 and then to 77.5 in 2018. However, relative accessibility was decreasing. In 2002, the index of relative accessibility was 81.4, which is a relatively high level, and then declined to 78.0 by 2009, and it dropped further to 67.3 in 2018 (**Figure 2**). The ITS analysis results showed that the 2009 health system reform significantly increased the index of absolute accessibility and the index of subjective perceptions. The index of relative accessibility had an immediate increase after 2009, but then continued to decline (Appendix S3 in the **Online Supplementary Document**).

### Index of affordability

**Figure 3:**
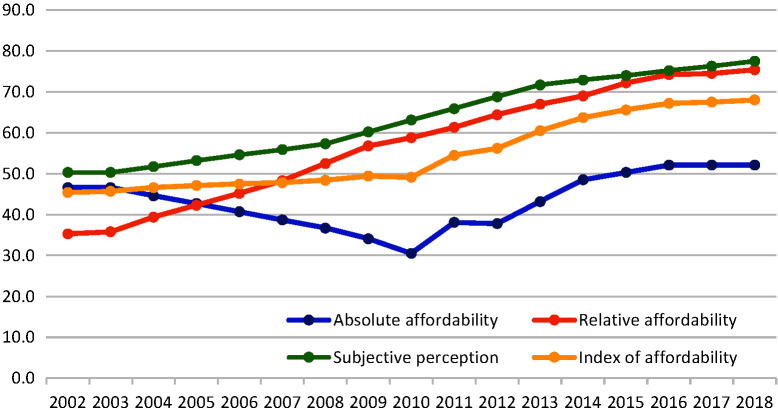
Change in Index of affordability in China (2002-2018).

Among the three components of affordability, the sub-index with the highest improvement was the relative affordability. In 2002, the relative affordability score was 35.3. It increased to 56.3 by 2009 and further increased to 75.4 in 2018. The trend in the index of absolute affordability was not linear. Between 2002 and 2010, this sub-index declined from 46.6 to 30.5. However, the trend began to reverse after 2010, increasing to 52.1 by 2018 (**Figure 3**). The ITS analysis results showed that the 2009 health system reform reversed the decline trend of the index of absolute affordability (*P* < 0.01), but slightly slowed down the increasing trend of the index of relative affordability (Appendix S3 in the **Online Supplementary Document**).

## DISCUSSION

### China’s progress towards UHC

This study applied the WHO indices for UHC to the Chinese context to measure and monitor China’s progress towards UHC. The results show that access to essential health services (absolute access) has increased since 2002. The strengthening of the health system, especially in terms of human resources and health financing, is the main driving force behind the improvement. Due to the expansion of medical education, China has trained an increasing number of physicians and nurses, which has led to an improved supply of health professionals [18,19]. China developed basic health insurance schemes for urban employees in 1998, for rural residents in 2003, and for urban residents in 2007 [20]. The majority of China’s population has been covered by these health insurance schemes. These increasing financial and human resources have encouraged the Chinese population to satisfy their unmet health needs and use more outpatient and inpatient services [10,21,22].

Despite the increase in absolute accessibility, the index of relative accessibility declined, reflecting that more patients are using hospital care rather than primary health care. PHC in China is struggling to attract qualified health workers and patients [23,24]. Although China is struggling to build a tiered service delivery system with a focus on PHC, patients still do not trust the quality of PHC services and instead swarm to large hospitals, even for minor illnesses [25]. In this situation, patients’ subjective feelings about difficulties in seeing a doctor are mainly about seeing a specialized doctor in a large hospital [23,26].

The index of affordability showed a different story. Before 2009, the public had severe complaints about the financial burden of medical services. The index of absolute affordability decreased between 2002-2009, reflecting that patients, especially low-income patients, were increasingly suffering from CHE. The main reason was that the uncontrolled escalation of medical expenses surpassed the growth rate of people’s income [27]. The health system reforms that started in 2009 focused on controlling medical expenses. Government financial investment, public hospital reform, PHC reform, and essential medicine and health financing reforms all contributed to the improvements in financial protection between 2009 and 2018 [4,28]. The trend in absolute affordability in this study exhibited a clear turning point in 2010. Recent publications have also reported this improved financial protection [26,29,30]. However, studies at the subnational level and of certain population groups suggest that the rate of catastrophic health expenses has not declined since 2011 [31,32]. More evidence is needed to validate the changing trends in financial risk protection in China.

Although there has been some improvement, the population in China still face a high financial burden when using medical services. The most recently available data showed that the incidence of CHE was 8.94% in 2016, and even higher for the low-income subpopulation [30,33]. However, for most OECD countries, the average CHE rate is approximately 5.8% [34]. China still has a long way to go in achieving UHC through financial protection, especially for low-income households, those in less developed areas, and those with chronic diseases.

Although the ITS analysis cannot fully explain the causal relationship between health system reform starting in 2009 and the changing trend in accessibility and affordability, the continuing declining trend of the index of relative accessibility implies that people’s preferences to use hospital care rather than PHC did has not changed after the health system reform. The sharp turning of the index of absolute affordability implies that the health system reform has contributed significantly to reduce the financial burden due to using health services.

### Discussion on the methodology

To our knowledge, this study is the first to adapt the WHO UHC indices to the context of a specific country. First, although the health system in China has made impressive improvements in recent decades, the fragmentation between hospital care and PHC has become a critical bottleneck for UHC progress. A UHC index that does not consider this key feature is not sufficient for suggesting further policy directions for achieving UHC. Second, since 2005, the public outcry about difficulties in accessing health services and about high medical expenses has largely guided China’s health system reform priorities. However, there is no consensus on the extent to which these issues have been addressed in the new round of health system reforms. The UHC indices in this study included people’s subjective feelings about the outpatient and inpatient services and showed the overall improvement in accessibility and affordability of health services since 2009. Third, UHC indices at the national level can enrich analyses by using more available data. This wide range of data used in this study allows us to comprehensively capture the dimensions of accessibility and affordability. It also provides a basis for analysing the changing trends in affordability and accessibility over the last 17 years and to compare these trends between provinces.

This study developed two independent indices for accessibility and affordability. These two aspects of UHC may be mutually reinforcing. On the one hand, increased use of outpatient and inpatient services inevitably lead to higher medical expenses and consequently a higher financial burden, while limited access to and underuse of health services often go hand-in-hand with a lower financial burden. On the other hand, a higher financial burden may prevent people from using health services, especially those with low incomes. This analysis can help provinces identify their specific challenges and propose prioritized policy options to improve health service accessibility and affordability.

One key limitation of this study is data availability [35]. Some indicators have missing values, especially at the provincial level. We used interpolation and extrapolation based on linear regression to impute these missing values [15]. As a result, indicators with more data points may have a greater weight in the calculation of the index. Equity and quality are critical dimensions of UHC, and we have included some indicators to measure equity. For example, we reported the CHE in both general population and low-income population (represents 20% of the population with the lowest income) to capture the equity in affordability, and the physicians’ education was used as a proxy indicator of quality in accessing health services. But for most of the indicators, data on the equity distribution across socio-economic groups were unavailable and therefore not included in the analysis. Future studies should include more valid data sources to strengthen the equity and quality components of the UHC indices, to validate the key findings on financial risk protection and to further monitor UHC progress at the national and subnational levels.

## CONCLUSION

China has made great progress in increasing the accessibility and affordability of health services since the health system reforms in 2009. However, integrating primary health care and hospital care and containing escalating medical expenditure to further reduce patients’ financial burdens are key challenges for strengthening the Chinese health system.

## Additional material

Online Supplementary Document
