# Supplementary material for: Measuring and evaluating progress towards Universal Health Coverage in China
Source: J Glob Health. 2021 May 1;11:08005. doi: 10.7189/jogh.11.08005 (PMC8088770; doi:10.7189/jogh.11.08005)
Supplement: Online Supplementary Document [file jogh-11-08005-s001.pdf]

Supporting information:

Appendix 1: Data transformation for constructing UHC indices in China

Index	Index component	Indicator	Calculation formulas	Ideal/minimum value and rationale	Data transformation	Imputation
**Accessibility**	**Absolute accessibility**	1. % of resident with access to the nearest health facilities within 15 minutes	-	**100%**	Not required	Linear interpolation and extrapolation (missing data: 2002, 2004-08, 2009-12, 2014-17)
		2. Number of physicians per 1,000 population	(Number of licensed physicians×1,000)/Number of residents	"Healthy China 2030" set a target to achieving **3** licensed physicians per 1,000 residents by 2030.[36]	Score = Number of licensed physicians per 1000 population/3.0, ⩾3 is 100 scores.	Not required
		3. % of physicians with bachelor's degree or above	-	**100%**	Not required	Linear interpolation (2003-04, 2006-08)
		4. Number of general practitioners per 10,000 population	(Number of GPs×10,000)/Number of residents	National health authorities set a target to achieving **5** GPs per 10,000 residents by 2030.[37]	Score = Number of GPs per 10,000 population/5.0, ⩾5 is 100 scores.	Linear extrapolation (before 2011)
		5. Number of outpatients visit per person per year	-	The most recent year median value of this indicator in OECD countries is **7.6**. [38]	Score = Number of outpatients visit per person per year/7.6, ⩾7.6 is 100 scores.	Linear extrapolation (2002-03)
		6. Annual hospitalization rate (%)	(Annual number of hospitalization×100/number of residents)×100%	Expert consultations set a target: **12%**	Score = Annual hospitalization rate/12%, ⩾12% is 100 scores.	Not required
		7. Coverage of essential public health services	Geometric mean of 15 indicators, see table 1.	**100%**	Not required	Not required
		8. % of PHC facilities equipped with essential medicine	-	**100%**	Not required	Not required (Not included)
		9. Coverage of basic health insurance schemes	[(Number of people enrolled in the Urban Employee Basic Medical Insurance, UEBMI + Urban Resident Basic Medical Insurance, URBMI + New Cooperative Medical System, NCMS)/number of residents] ×100%	**100%**	Not required	No imputation
	**Relative accessibility**	10. % of hospitalization within the county	-	National health authorities set a target to achieving **90%** of hospitalization within the county by 2020.[39]	Score = % of hospitalization within the county/90%, ⩾90% is 100 scores.	Not required (Not included)
		11. % of outpatient service utilization at PHC level	(outpatient service utilization at PHC level/ all outpatient service utilization) ×100%	**70%** was regarded as the target value since WHO argued that PHC could cover 70% of health need. 30% was the worst scenario (the 2.5th percentile of this indicator at provincial level).	Score=[(% of outpatient service utilization at PHC level -70%)/(30%-70%)]×100%, ⩾70% is 100 scores.	Linear extrapolation (2002-2008)
		12. % of patients	(number of patients recommended to hospitalization	**0%**	Score = (1-% of patients	Linear interpolation and

		recommended but not using inpatient service	but not using inpatient service /number of patients recommended to hospitalization) ×100%		recommended but not using inpatient service) ×100%	extrapolation (2002, 2004-08, 2009-12, 2014-17)
	**Subjective perception on accessibility**	13. Patients' satisfaction with outpatient services	(number of outpatient care patients in the past two weeks satisfied with their experiences /number of all outpatient care patients in the past two weeks) ×100%	**100%**	Not required	Linear interpolation and extrapolation (2002, 2004-08, 2009-12, 2014-17)
		14. Patients' satisfaction with inpatient services	(number of inpatient care patients satisfied with their experiences in the past one year /number of all inpatient care patients in the past one year) ×100%	**100%**	Not required	Linear interpolation and extrapolation (2002, 2004-08, 2009-12, 2014-17)
**Affordability**	**Absolute affordability**	15. % of catastrophic health expenditure	Catastrophic is defined if household expenditure on health/household nonfood consumption > 40%	**1%** was regarded as the target value and **28%** was the worst scenario since the World Bank monitoring report in 2015 found that 1% and 28% was the 2.5th and 97.5th percentile of this indicator at a global level, respectively.[1]	Score = [(incidence of catastrophic health expenditure-28%)/(1%-28%)] × 100%	Linear interpolation and extrapolation (2002, 2004-08, 2009, 2013, 2015, 2017)
		16. % of catastrophic health expenditure among low income group	The incidence of catastrophic health expenditure among the households at the lowest quantile of the expenditure's distribution (0-20th).	See the indicator 15.	See the indicator 15.	See the indicator 15.
	**Relative affordability**	17. % of medical expenses covered by health insurance	$\sqrt[3]{}$(% of medical expenses covered by UEBMI ×URBMI×NCMS)	National health authorities set a target to achieving **90%** of medical expenses covered by health insurance.	Score = (% of medical expenses covered by health insurance/ 90%) × 100%, ⩾90% is 100 scores.	Linear interpolation and extrapolation (2002, 2004-07, 2009-12)
		18. % of out of pocket payment in total health expenditure	(out of pocket payment, current price / total health expenditure, current price)×100%。	The most recent year median value of this indicator in OECD countries is **17.15%**.[38]	Scores = (100%-% of out of pocket payment in total health expenditure)/(100%-17.15%) ×100%, ≤17.15% is 100 scores.	Not required
		19. % of total health expenditure in GDP	(total health expenditure, current price / GDP, current price)×100%。	The most recent year median value of this indicator in OECD countries is **8.87%**.[38]	Score = (% of total health expenditure in GDP /8.87%) ×100%, ⩾8.87% is 100 scores.	Not required

Note:

GPs: general practitioners. PHC: primary health care. OECD: Organization for Economic Co-operation and Development. WHO: World Health Organization. GDP: gross domestic product.

Appendix 2: Sensitivity analyses

Original Indices

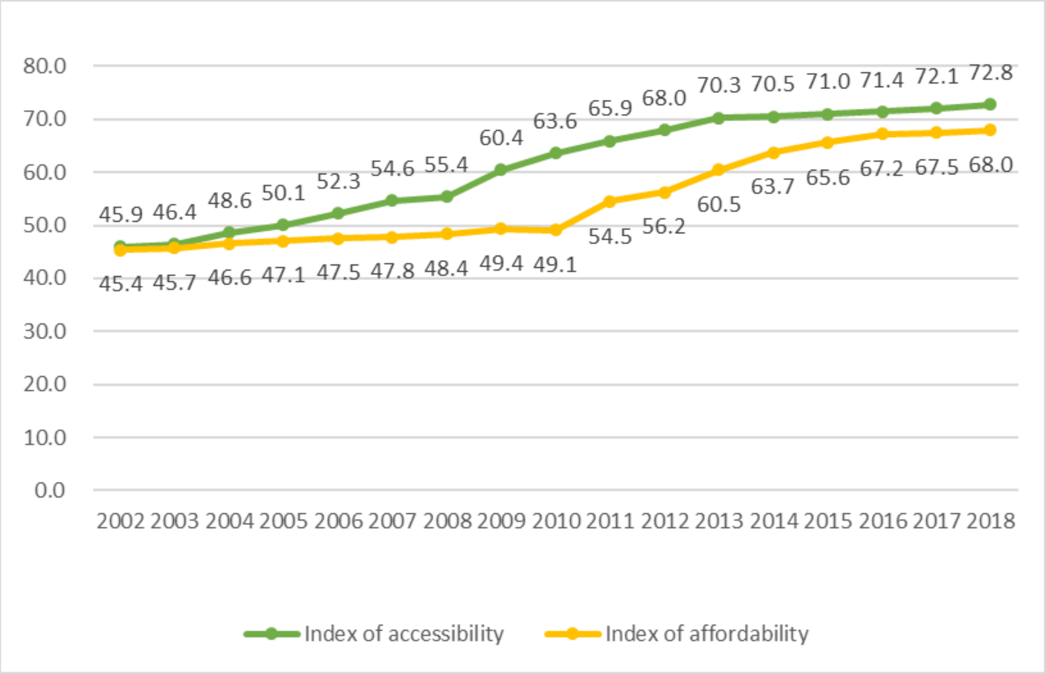

Scenario 1: re-calculating with geometric mean of all indicators (no weight)

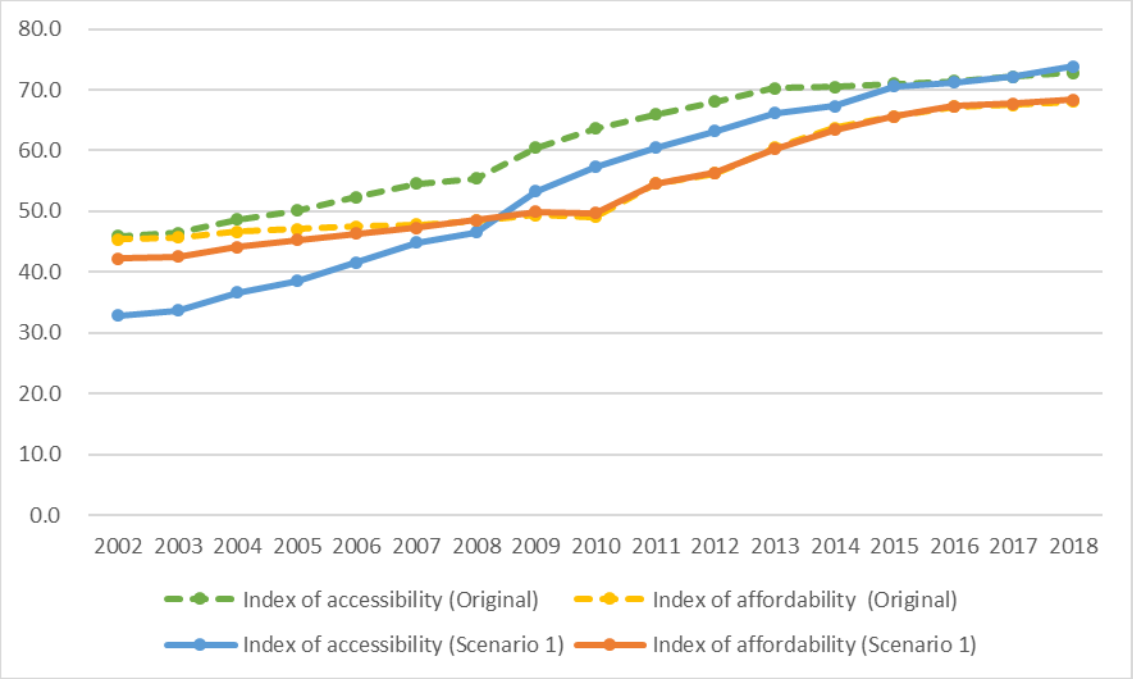

Scenario 2: re-calculating with arithmetic mean

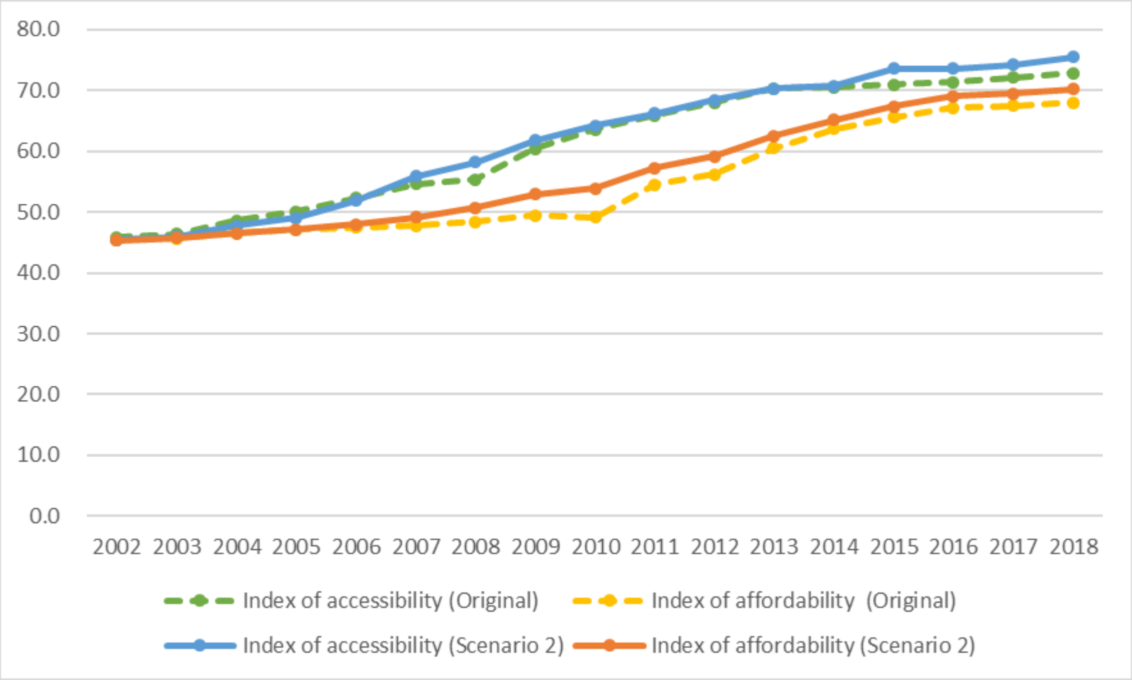

Scenario 3: re-calculating without imputation

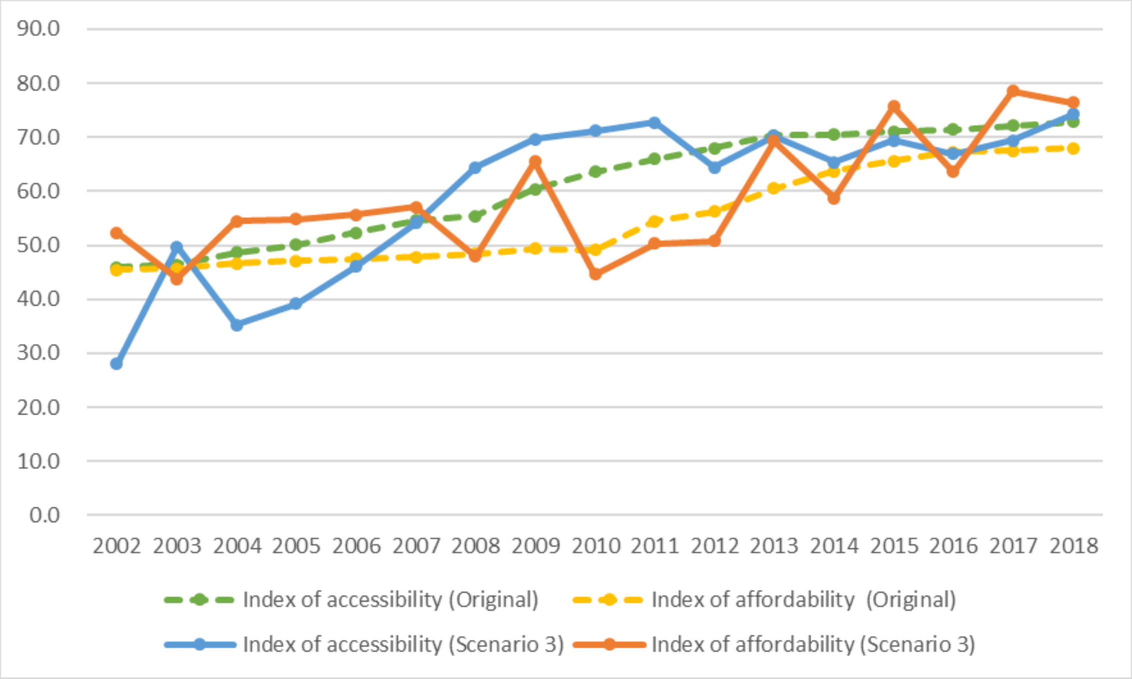

Scenario 4: re-calculating and deleting one indicator at a time

Index of accessibility:

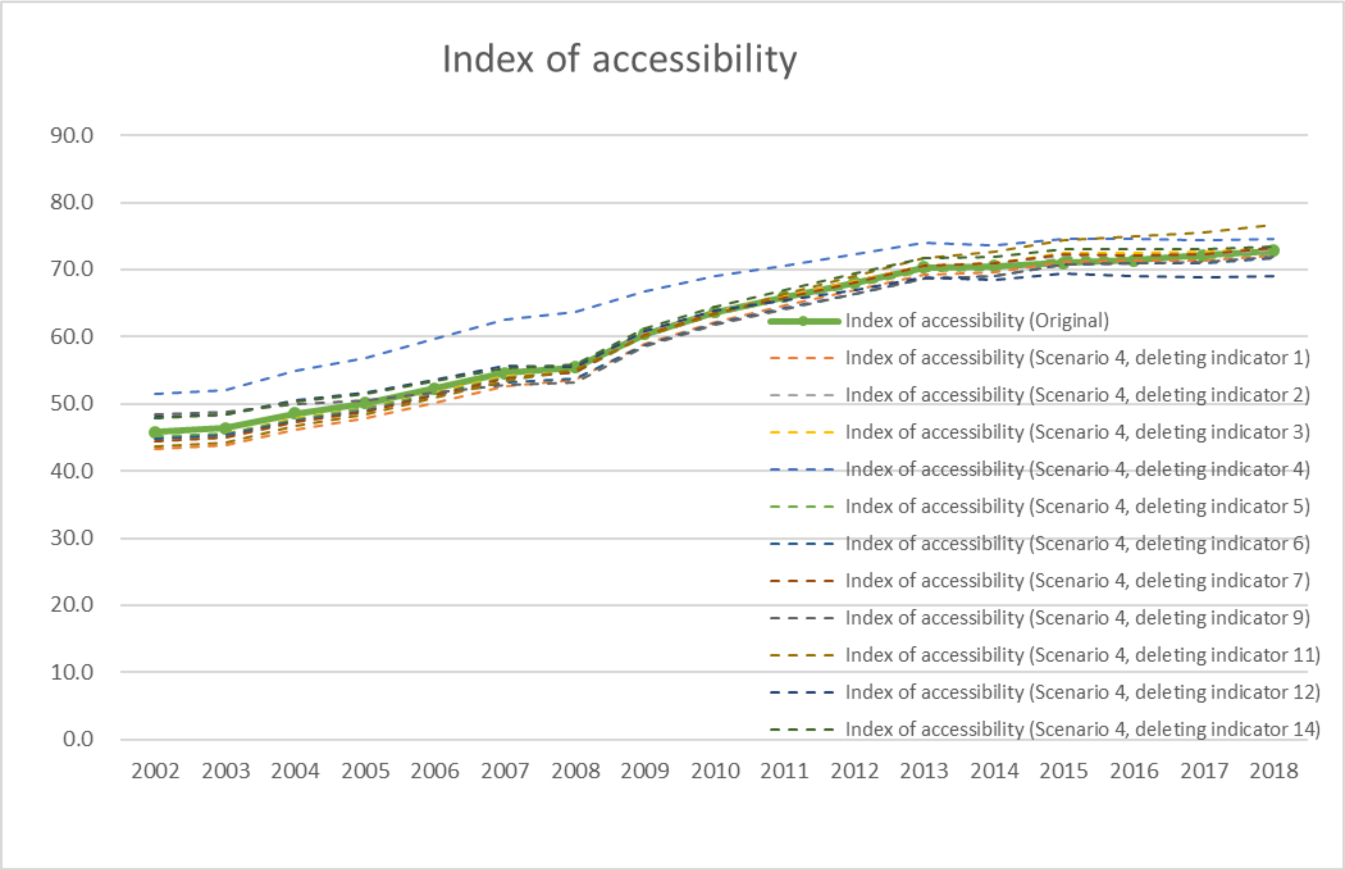

Index of affordability:

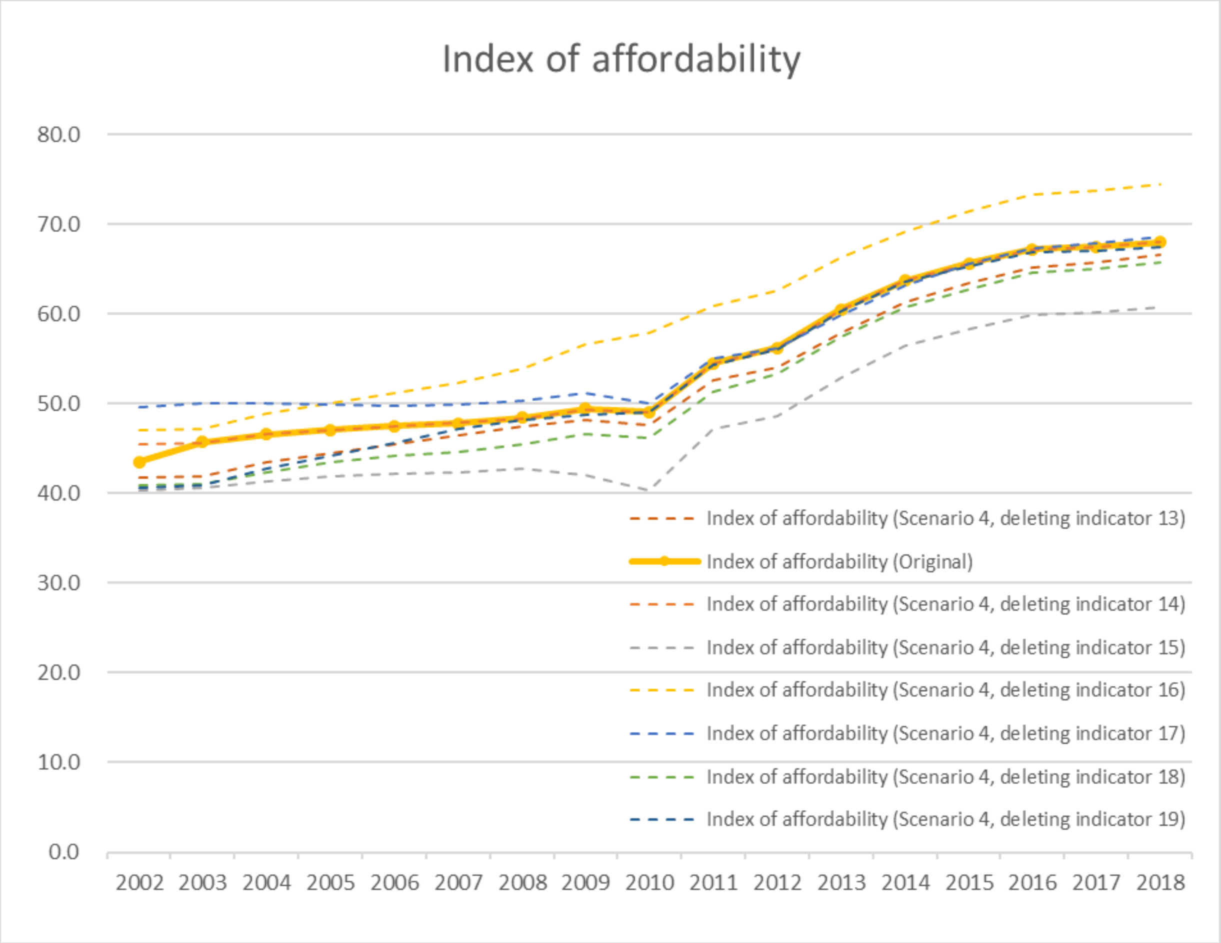
Index of affordability
80.0
70.0
60.0
50.0
40.0
30.0
20.0
10.0
0.0
2002 2003 2004 2005 2006 2007 2008 2009 2010 2011 2012 2013 2014 2015 2016 2017 2018
Index of affordability (Scenario 4, deleting indicator 13)
Index of affordability (Original)
Index of affordability (Scenario 4, deleting indicator 14)
Index of affordability (Scenario 4, deleting indicator 15)
Index of affordability (Scenario 4, deleting indicator 16)
Index of affordability (Scenario 4, deleting indicator 17)
Index of affordability (Scenario 4, deleting indicator 18)
Index of affordability (Scenario 4, deleting indicator 19)

Appendix 3: Interrupted time-series analysis results

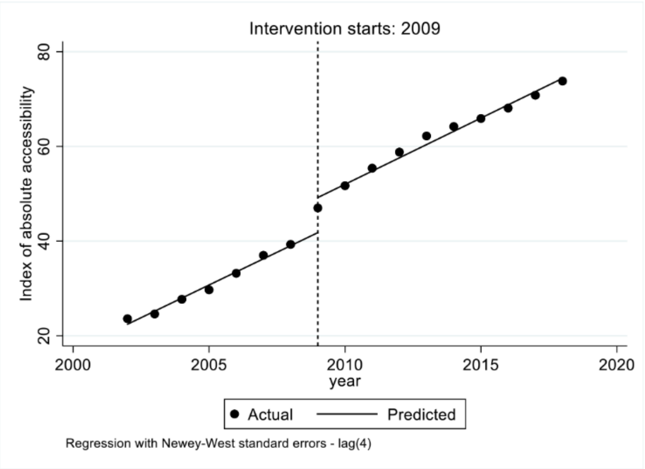

Absolute accessibility

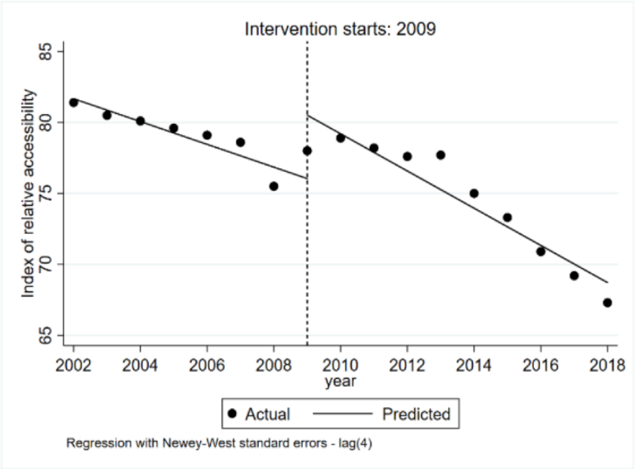

Relative accessibility

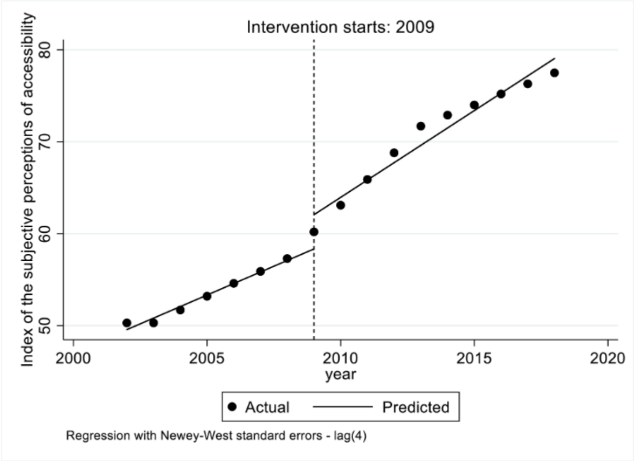

Subjective perceptions

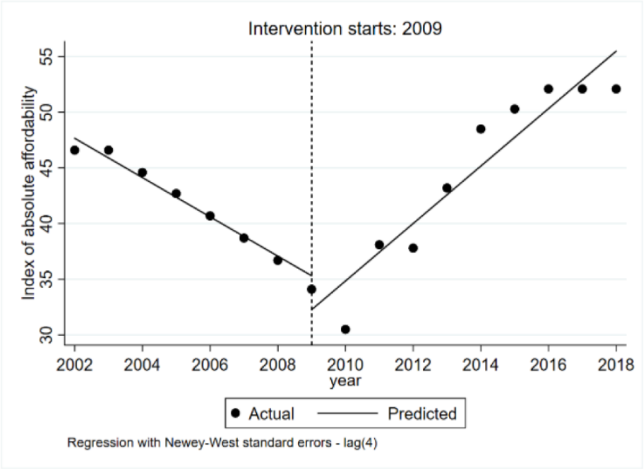

Absolute affordability

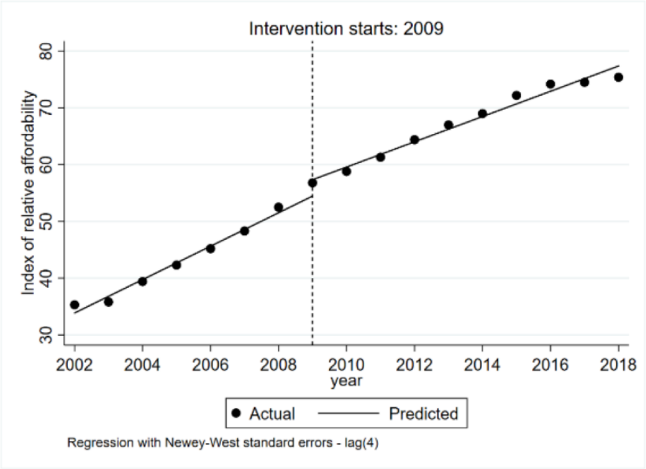

Relative affordability

	**Absolute accessibility**	**Relative accessibility**	**Subjective perceptions**	**Absolute affordability**	**Relative affordability**
Pre-2009	2.76*** (0.14)	-0.80***(0.13)	1.25***(0.07)	-1.76***(0.11)	2.94***(0.15)
2009	5.68***(1.31)	4.47*** (1.03)	3.72***(1.17)	-3.05**(1.21)	2.89**(1.05)
Post-2009	0.03(0.17)	-0.51*(0.28)	0.63***(0.18)	4.34***(0.29)	-0.72***(0.18)

Notes: Coefficients from ITS are reported as the effect of the 2009 health system reform on the outcome variables which are listed in the columns. The number of observations is 17 for each of the ITS analysis; Standard errors are reported in parentheses; ***p<0.01; **p<0.05; *p<0.1.

Interpretation of the results: take absolute accessibility for example. Before 2009, the annual increase rate of the index of absolute accessibility was 2.76 per year. The index of absolute accessibility increased 5.68 the first year after the 2009 health system reform. The annual increase rate of the index of absolute accessibility after 2009 was 0.03 higher than the annual increase rate before 2009, but the statistical test was insignificant due to the limited number of observations.

Appendix 4: Spatial pattern of Index of affordability and Index of accessibility scores in 2018

Panel A: Joint visualization of indices of accessibility and affordability in 25 provinces in 2018

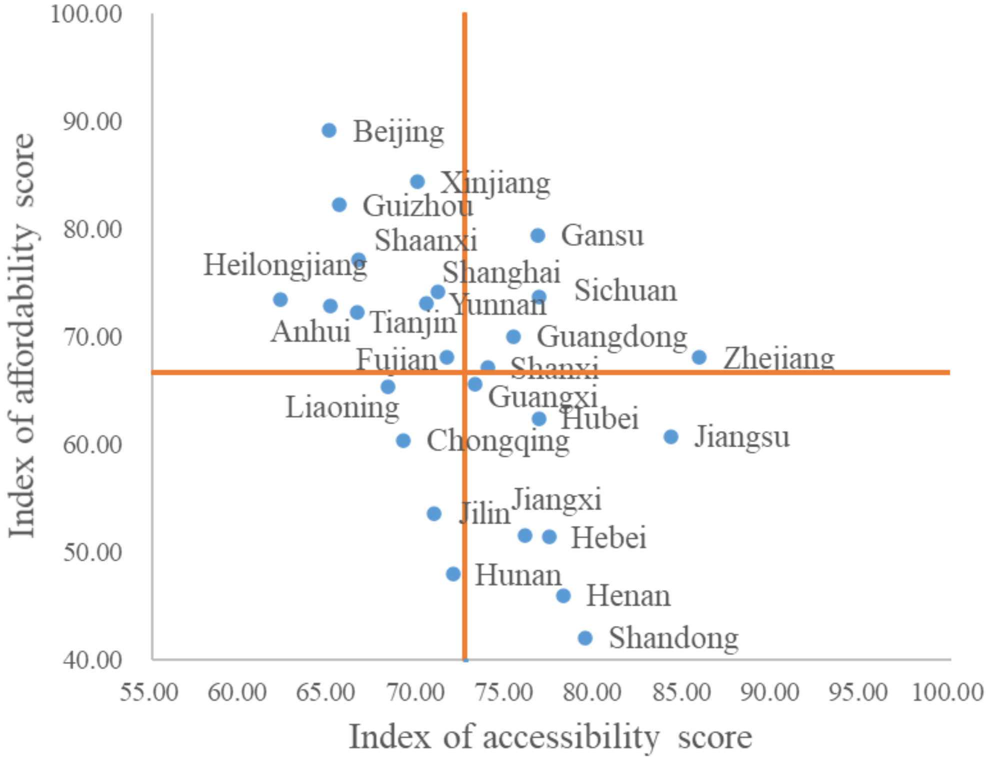

Note: Five provinces and regions of mainland China (Inner Mongolia, Hainan, Tibet, Qinghai, and Ningxia) were excluded from the index calculations due to data unavailability.

Panel B: Index of accessibility scores

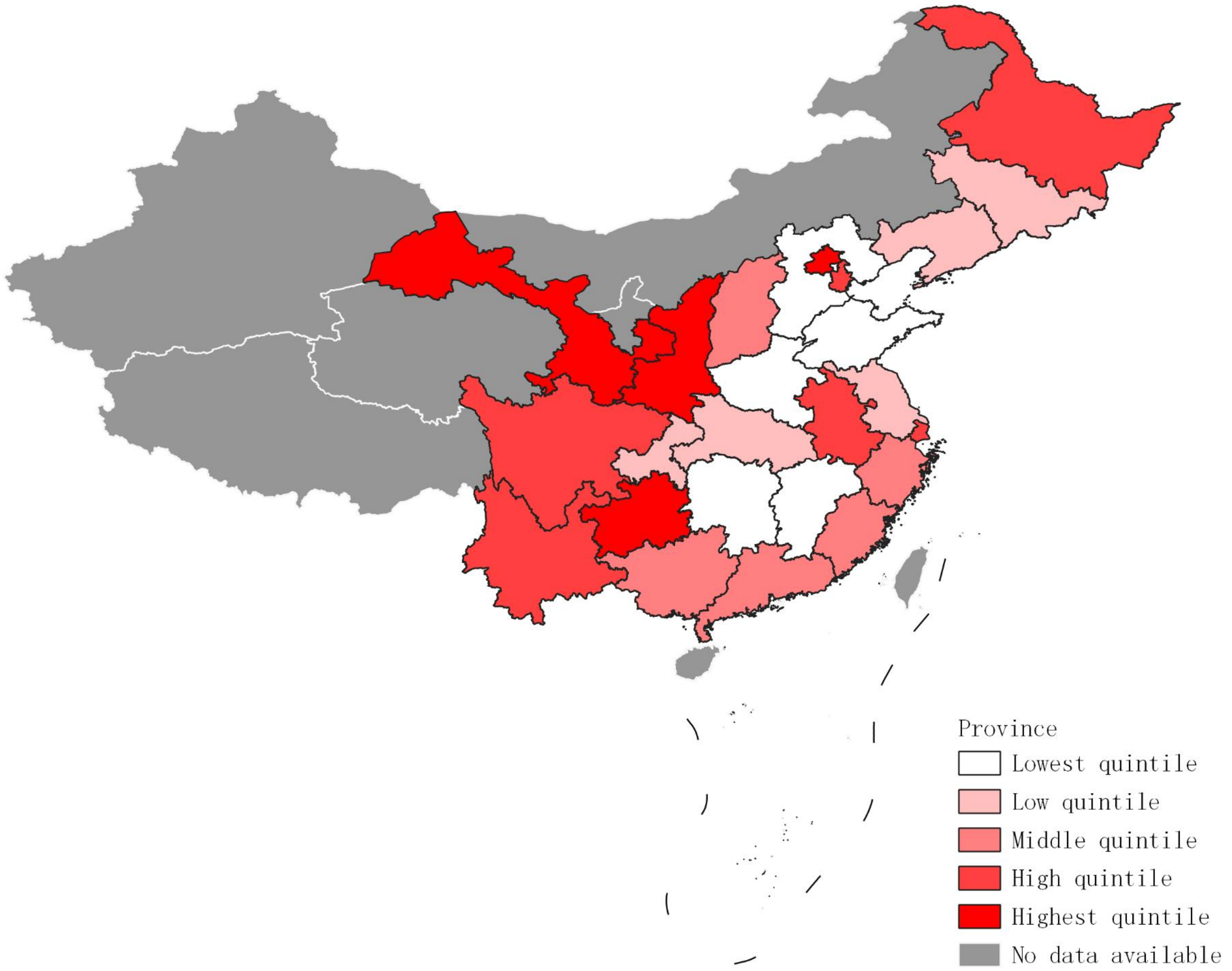

Panel C: Index of affordability scores

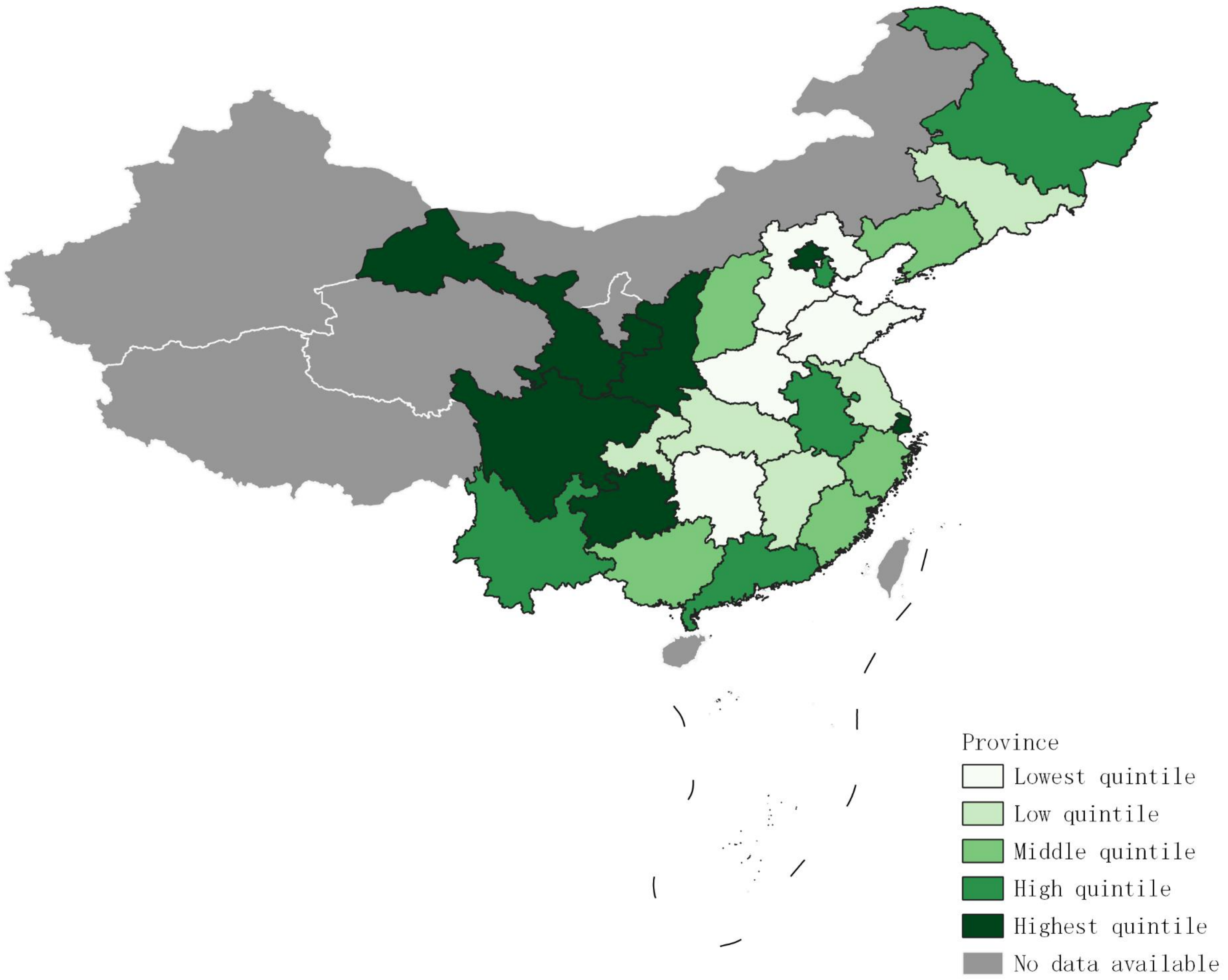

Note: Six provinces and regions of mainland China (Inner Mongolia, Hainan, Tibet, Qinghai, Xinjiang, and Ningxia) were excluded from the index calculations due to the data availability.

Appendix 5: abstract in Chinese

评价中国全民健康覆盖的进展情况：衡量中国在“看病难”、“看病贵”方面取得的进展

摘要

背景

本文旨在开发中国版的全民健康覆盖指数，并衡量中国在全国和省级层面上实现全民健康保险的进展。

方法

我们在专家协商的基础上选定了 19 个指标，以构建衡量全民健康覆盖的可及性（衡量中国的“看病难”程度）和可负担性（衡量中国的“看病贵”程度）的指数。数据来自中国卫生健康统计年鉴、全国代表性调查和医改监测数据。可及性指数包括绝对可及性（基本卫生服务的可及性）、相对可及性（住院治疗的可及性）和人们的主观感受；可负担性指数包括绝对可负担性（灾难性卫生支出的发生率）、相对可负担性（卫生支出的构成）和人们的主观感受。

研究结果

在 17 年的观察期内中，中国可及性指数和可负担性指数都显示出稳步增长。绝对可及性的改善最为显著（从 2002 年的 23.6 提高到 2018 年的 73.8），而相对可及性指数则从 2002 年的 81.4 下降到 2018 年的 67.3。绝对可及性指数从 2002 年的 46.6 大幅下降到 2010 年的 30.5，之后开始反弹，2018 年达到 52.05。与此同时，绝对可达性指数持续上升，从 35.3 上升到 75.4。

结论

自 2009 年医疗卫生体制改革以来，中国在提高卫生服务的可及性和可负担性方面取得了巨大进展。然而，整合基层卫生和医院医疗服务，控制不断攀升的医疗支出，和进一步减轻患者的经济负担，将是进一步加强中国卫生系统的关键挑战。

关键词：全民健康覆盖、可及性、可负担性、监测与评估、中国
